# What can we learn from the history of male anorexia nervosa?

**DOI:** 10.1186/s40337-014-0036-9

**Published:** 2014-12-31

**Authors:** Chengyuan Zhang

**Affiliations:** Imperial College London; South Kensington Campus, SW7 2AZ Exhibition Road, London, UK

**Keywords:** Anorexia nervosa, Eating disorder, History of psychiatry

## Abstract

The eating disorders literature has focussed on females and little is known of the male experience. The overall image this has generated suggests a young woman in conflict with socio-cultural pressures which associate thinness with beauty. Historical studies have examined anorexia nervosa from an entirely female focus while ignoring how diagnostic categories have shaped approaches to the male body. This paper will track the case of the male with anorexia nervosa through changing theories of causation and treatment approaches, from when the condition first emerged in 1873 to the present. In doing so, we gain a valuable new insight into how anorexia nervosa has been historically gendered and the far-reaching implications this has had for diagnosis and treatment of the male sufferer.

Similarities between the sexes helped to establish male anorexia as a distinct category. However, this shifted focus away from important differences, which have yet unexplored implications in the assessment, diagnosis and management of disordered eating. Throughout history, there has been constant pressure to give a precise definition to anorexia nervosa, despite being fraught with medical uncertainties. This has resulted in inevitably harmful generalisations rooted in the dominant epidemiology. This paper reveals that anorexia nervosa is a truly global phenomenon which cannot be adequately constructed through exclusive studies of the female. There is consequently a pressing need to address the dearth of research examining eating disorders in males.

## Background

Anorexia nervosa is currently viewed as an almost exclusively female disorder and women account for 90% of those diagnosed. However, an estimated 3 in 10 cases occur in males who are often overlooked, underdiagnosed and understudied in research [1]. Ever since the clinical term was first coined by Sir William Gull in 1874, anorexia nervosa has been bound to an inherently female nosology. Over-simplistic explanations throughout history, from hysteria, ovarian failure and oral impregnation to the thin female ideal, have resulted from the assumption of a unilaterally female epidemiology. These have produced treatment approaches which, throughout history, have persistently neglected to consider the important differences between men and women struggling with disordered eating.

### A historical perspective

In an address to the Clinical Society on ‘Anorexia Hysterica (Apepsia Hysterica)’ in 1873, Sir William Gull described a disease ‘occurring mostly in young women between the ages of fifteen and twenty-three, and characterised by extreme emaciation’ [2]. Gull made no mention of males and despite introducing the term ‘Anorexia Nervosa’ a year later, the condition became intimately tied to hysteria, an inherently female diagnosis [3]. The main treatment approach was separation from the family, as the ‘thin-blooded emotional’ female was seen as ‘a vampire who sucks the blood of the healthy people about her’ [4]. Yet, while still dominant in the years before and after the First World War, this orientation would be challenged from two different quarters: new psychiatric approaches, partly stemming from interest in war-related conditions, notably shell-shock, and in the socio-genic role of the family, began to explore ‘male anorexia’; and the new science of endocrinology sought to establish a biochemical basis for anorexia, igniting dynamic debate over the existence of male forms of the disorder.

The first medical definitions of anorexia nervosa left little room for identifying male sufferers. Increasingly, however, anorexia nervosa was being reframed as a psychiatric problem linked to dysfunction within the family. This made it possible to conceive of a male form of anorexia. In 1931, a group of eminent British physicians attributed male cases to ‘an authoritarian father, and a weak (crushed) mother’ and being ‘homosexualist’ [5]. This family-oriented explanation derived from preconceptions of hysteria and a focus on the mother-daughter relationship within families of adolescent girls. However, there was a lack of knowledge surrounding male family dynamics which has persisted to the present [6]. Despite this, males with anorexia nervosa were viewed as effeminate homosexuals who, like their female counterparts, needed to be isolated from their families.

One of the first challenges to earlier models centred on hysteria came in 1914 through descriptions of Simmonds’ disease, an endocrine disorder of the pituitary gland which closely resembled anorexia nervosa, with amenorrhoea as a key symptom of both conditions [7]. Physicians searched for an endocrine cause of amenorrhoea, erasing male cases altogether by connecting anorexia nervosa with ‘primary ovarian failure’ [8]. Oestrogen injections were given to stimulate menstrual periods, generating a focus on amenorrhoea which became embedded within diagnostic categories until DSM-5 [9]. However, during the course of successive DSM revisions, studies have repeatedly questioned the diagnostic relevance of amenorrhoea, demonstrating that it reflects nutritional status rather than psychopathology [10-12]. Furthermore, the absence of an analogous criterion for men built a gender bias into the first definitions of the disorder.

By the 1940s, a new approach rooted in Freudian psychoanalysis had seized the ground. Anorexia nervosa was framed as a female sexual neurosis where food was associated with impregnation and obesity with pregnancy [13]. Amenorrhoea was now seen as a direct psychological consequence of pregnancy fantasies and constipation symbolised the child in the abdomen [13]. Despite aims of developing a singular explanation for anorexia nervosa, psychosexual approaches focussed on female sexual dysfunction, which was targeted aggressively by psychotherapy. Oestrogen treatment continued, not to compensate for any ovarian failure, but for ‘ovarian deficiencies’ linked to the ‘anorectic’s psychic state’ [7].

However, neither oestrogen injections nor psychotherapy produced positive results, and these ideas were increasingly challenged [14]. Hilde Bruch championed new approaches to the disorder in the 1960s, finding the same core features in male and female cases, including an endocrine dysfunction ‘gauged by urinary output of testosterone’ comparable to amenorrhoea [15,16]. These findings, embodied by DSM-III in 1980, redefined anorexia nervosa, with ‘distortion of body image’ and ‘fear of ugliness’ as its primary features [15,17]. However, attention to similarities between the sexes shifted focus away from important differences. As a result, treatment approaches generated exclusively by studies on female subjects went unchallenged when applied often inappropriately to their male counterparts.

Subsequently, second-wave feminism around the 1980s framed anorexia nervosa as evidence of a misogynistic society which objectified the female body and devalued female experiences. This helped to establish a psychotherapeutic approach targeting female body image issues within ‘a culture that praises thinness and fragility in women’ [18,19]. To this day, psychotherapy does not comprehensively address the disorder in males. Feminist analyses have disregarded the male as an unlikely candidate for the diagnosis. As psychologist Julie Hepworth argues, male cases provided ‘a discursive problem because the dominant explanation of anorexia nervosa specifically links it with an ideology of femininity’ [20]. In light of feminist socio-cultural framing, males are still often excluded from clinical research, despite the same core psychopathology in both sexes. Additionally, the widely used Eating Disorders Inventory-3, developed from and validated in female populations to predict eating disorders, has drawn criticism for its lower reliability in men [21]. Given men are already underdiagnosed, there is a pressing need to construct a validated male-specific assessment tool.

### Lessons for the future

Insufficient attention has been given to reasons why males develop the disorder. Feminists have framed the disorder as one which is socially produced by patriarchical social structures; what happens to non-dominant males within a patriarchy is never specified. While gender has been a key ‘category of analysis’ [22], it has been used asymmetrically, concentrating on how anorexia nervosa has been gendered as ‘female’ while ignoring how it shaped approaches to the male body. Within this framework, feminists have assumed that men are also subject to many of the same social pressures of aestheticism as women, without separate study of the male subject [23].

Yet, feminist concern with how gender shapes social and medical categories is critical to better understanding male anorexia nervosa and, crucially, to its acceptance as a condition. Women and men are ‘defined in terms of one another’ and anorexia nervosa cannot therefore be adequately constructed by entirely separate study of the female [22]. This approach has historically created a string of stigmatising suppositions which have all failed to fully encapsulate the condition. Early female victims were cruelly force-fed to combat an emaciation thought to result from emotional excesses or inappropriate sexual fantasies of oral conception and pregnancy. Men were either missing from medical discourses or feminised as homosexuals. Whilst homosexuality is currently a specific risk factor for anorexia nervosa in men, ‘most men with body image disorders are straight and most gay men do not have body image disorders’ [24,25]. Diagnostic categories have repeatedly been created out of assumptions, supported by examples which affirm the definition whilst ignoring exceptions which the dominant rule cannot explain.

Currently, this approach stigmatises sufferers as young women trapped in a vicious cycle to achieve a thin-ideal. This view has fundamentally shaped the identity and status of male sufferers who bear the added burden of a feminised diagnosis. Yet, the literature reveals crucial differences between male and female experiences of eating disorders which may have unexplored treatment implications [26]. It has recently been suggested that muscle dysmorphia, which shares a similar concern for body image, food intake and exercise, may be classified as an eating disorder with a predominantly male epidemiology [27]. The similarities that can be drawn with anorexia nervosa emphasise a need to understand male experiences of body image disturbance and the impact constructions of masculinity and femininity have on the sufferer [28].

Whilst anorexia nervosa and bulimia nervosa are strongly associated with the female sex, several epidemiological studies have found binge eating to be equally common in men and women [29]. Emerging evidence also suggests that binge eating may be more debilitating for men [30]. However, few studies on binge eating have included men despite this equal gender distribution. The way in which anorexia nervosa has been historically constructed as a ‘woman’s illness’ has enduring implications in the way we currently view eating disorders in general. At present, men are less likely to receive treatment and are treated for fewer days [31]. We must therefore decouple the experience of eating disorders from feminised cultural imagery in order to break down the barriers men face in recognising their symptoms and seeking help [32]. There is a need to explore specifically and in detail what it is like to inhabit a male body in the context of anorexia nervosa. Only then can we appropriately clarify fact from fiction – what anorexia nervosa really is and what it is not – to improve the way we assess, diagnose and treat all groups of people.
